# Laboratory tests and compliance of dermatologic outpatients

**DOI:** 10.12688/f1000research.2-206.v1

**Published:** 2013-10-07

**Authors:** Won Ung Shin, Yoo Sang Baek, Tom Joonhwan Kim, Chil Hwan Oh, Jaehwan Kim

**Affiliations:** 1 Department of Dermatology, College of Medicine, Korea University, Seoul, Korea, South; 2 Robert H. Smith School of Business, University of Maryland, College Park, MD 20742, USA; 3 Laboratory for Investigative Dermatology, The Rockefeller University, New York, NY 10065, USA

## Abstract

Laboratory tests, including blood tests and urine analysis, are frequently performed in the dermatology outpatient clinic, but doctors often do not consider the cognitive or psychological effect of the examinations. Based on terror management theory, we hypothesized that performing laboratory tests increases the patient’s fear of mortality, and therefore has a positive effect on the patient’s attitude toward the doctor’s recommendations and willingness to accept them. The study employed a single factor between-subjects design, using a questionnaire completed by the patients. One group consisted of patients who had undergone laboratory tests 1 week before the survey, and the other group consisted of patients who had not undergone a laboratory test. Although the differences between two groups were not statistically significant, the patients who had laboratory tests had tendency to show even lower positive attitude toward the doctor’s recommendations and less intention to follow the recommendations. In contrast to our hypothesis, performing laboratory tests does not subliminally increase patients’ fears or anxieties about their disease or their compliance with doctors’ recommendations.

## Introduction

Compliance in health care has been defined as the extent to which a person’s behavior coincides with health-related advice ^1^. Numerous factors have been suggested to influence patient compliance, including individual characteristics, illness type and severity, medication complexity, and emotional status ^2^. Adherence to treatment of the patients with several chronic diseases such as diabetes, glaucoma, multiple sclerosis and psychological problems was studied in various trials that emphasized the influence of psychological components ^3– 5^. However, most surveys of patient compliance in dermatology have focused on dermatological diseases and types of treatment ^6, 7^.

In this study, we hypothesized that the examinations performed in dermatologic clinics may affect patient compliance. In a dermatologic clinic, laboratory tests such as blood and urine tests are one of the main examinations along with skin biopsy. Laboratory tests are usually performed to diagnose systemic disease associated with skin lesions or to monitor patient’s vital organ functions. Dermatologists usually focus on the results of laboratory tests without considering the psychological impact of the tests.

One of the studies on patient compliance in dermatology by Kim *et al.* ^8^ examined the psychological influence of skin biopsy based on terror management theory (TMT). TMT proposes that awareness of death is a critical motivating force in human behavior ^9^. The theory states that people cope with fear against death through sustaining faith in cultural worldviews, and get self-esteem by pursuing to the standards of values provided by the cultural worldviews. A cultural world-view means ‘humanly created and transmitted beliefs about the nature of the reality shared by groups of individuals’ ^10^. By following cultural values and engaging in culturally prescribed behaviors, one can pursue the meaning of existence and perpetuity ^10– 12^. The study by Kim *et al.* ^8^ showed that receiving a skin biopsy led patients to have a greater tendency to follow a doctor’s recommendations.

Most patients visiting dermatologic clinics are not concerned about systemic disease, vital organ dysfunction or mortality, unless they have chronic skin diseases or skin cancers. However, we hypothesized that performing laboratory tests subliminally reminds patients of systemic disease, vital organ dysfunction or mortality. Based on TMT, laboratory test could increase patients’ death-related thinking and might increase their willingness to accept the recommendations of doctors, whose authority is acknowledged by culture. We investigated the relationship between performing laboratory tests and acceptance of a doctor’s recommendations. In addition, the effects of the patient’s trust toward doctors, authoritarian personality, and mood on medical compliance were also studied.

## Material and methods

### Participants

Among the patients that attended the dermatology clinic in Korea University Guro Hospital (Seoul, South Korea) between 1 July 2011 and 31 August 2011, a total of 100 first time visitors were requested to fill out the survey. Written informed consent was obtained from all participants in this study. A total of 45 male patients and 55 female patients were included in the study. A group of 50 patients underwent laboratory tests, while the other group of 50 patients did not have any laboratory tests. Laboratory tests included urine analysis and blood tests such as: complete blood count, liver function test, blood urea nitrogen analysis, and creatinine analysis. We included patients aged between 18 and 65 years old and excluded patients who had a history of psychological disorders, -had undergone other tests besides the above mentioned laboratory tests (such as a skin biopsy or a patch test), or who had undergone procedures such as laser treatment or cryotherapy.

### Survey

The patients were asked to complete the survey within two weeks on their second visit to the clinic. The survey measured their trust towards doctors, authoritarian personality, current mood, attitude toward the doctor’s recommendation, and intention to follow the recommendations. Participants were asked to indicate the extent to which they agree with a given statement on the survey by using a seven-point scale (strongly disagree, disagree, disagree somewhat, undecided, agree somewhat, agree, strongly agree). The full questionnaire is available as supporting data.

### Trust toward doctors

Several studies have measured patient’s trust in physicians using scales ^13, 14^, and we modified those scales so they included 5 statements to measure trust towards doctors: “I believe that doctors perform: 1. Perform only medically necessary tests and procedures, 2. keep personally sensitive medical information private, 3. perform necessary medical tests and procedures regardless of cost, 4. put my health and well-being above keeping down the health plan’s cost (reverse scale), and 5. I trust the doctors’ judgment when it comes to my medical care”. In case of fourth state reverse scale was used, and average of patients’ scale to these five questions was used.

### Authoritarian personality

The grades of authoritarianism are 1. adheres strongly to conventional moral values, 2. submissive to established authorities, and 3. willingness to be aggressive toward others if they are perceived as unconventional or threatening ^15^. We hypothesized that the grade of authoritarian personality, together with the amount of trust towards doctors, might influences patients compliance with medical treatment. K. Min ^16^ has developed standards for measuring Korean authoritarian personality grades, and the scale consist of 35 questions measuring conventionalism, authoritarian obedience, authoritarian aggressiveness, anti-introspectionism, stereotypical thinking, belief in power, cynicism, and sexism. Based on this scale, we randomly selected four items measuring authoritarian obedience, and average of patients’ scales to these questions was used.

### Mood

We considered that the mood of the patients during the survey was a potential confounder affecting patient compliance, and patients who underwent laboratory tests might feel differently compared to patients who did not. To measure patient mood, they were asked to answer the twenty questions measuring their current mood (PANAS-X(17)). This measurement consists of 10 positive feelings ( *active, alert, attentive, determined, enthusiastic, excited, inspired, interested, proud, strong*) and 10 negative feelings ( *afraid, scared, nervous, jittery, irritable, hostile, guilty, ashamed, upset, distressed*). Average of patients’ scales to these twenty questions was used.

### Attitude toward recommendation and intention to accept recommendations

In the last part of the survey, patients read a scenario: “You have a severe and chronic skin disease, and your doctor recommends a newly developed treatment method for you. He tells you that this method can reduce treatment time, but it is more expensive than traditional treatment and safety is not fully guaranteed”. After reading the scenario, the attitude toward the scenario was measured using three 7 scale questions that were used in previous marketing studies ^18^. Average of patients’ scales to these three questions was used.

As another dependent variable, the intention to accept recommendations was measured by answering the following two statements: “I will follow the recommendations of the doctor,” and “There is a possibility of following recommendations of the doctor”. The patients responded on a seven-point scale (strongly disagree, disagree, disagree somewhat, undecided, agree somewhat, agree, strongly agree). Average of patients’ scales to these two questions was used.

## Statistical analysis

SPSS software (Version 15.00, SPSS Inc, Chicago, IL, USA) was used to conduct the statistical analyses and *p*-value less than 0.05 was considered to be statistically significant. Data analysis consisted of the unpaired *t*-test, two-way analysis of variance (ANOVA) test and correlation analysis.

## Results

### Demographic analysis

Forty five (45%) male patients and fifty five (55%) female patients completed the survey. Each group (laboratory tests vs. no laboratory test) consisted of 50 patients, and the mean age of the groups was 37.42 years for the laboratory test group (range 19–65) and 39.56 years for the group not receiving laboratory tests (range 19–64). The dermatologic diagnoses were about evenly distributed over the two groups and were classified into cutaneous vascular disease, diseases of the skin appendages (hair and sebaceous gland), eczematous disease, erythema or urticaria, infectious disease, papulosquamous disease, pigment anomalies, pruritus, and tumors. Table 1 details the subject characteristics.

**Table 1.** Patient demographics.

	Laboratory test	No laboratory test
n/N(%)	50/100(50)	50/100(50)
M/F ratio	0.79(22/28)	0.85(23/27)
Mean age	37.42(19–65)	39.56(19–64)
Diagnosis		
Cutaneous vascular disease	3	1
Diseases of the skin appendages	5	8
Eczematous disease	9	14
Erythema or urticaria	19	20
Infectious disease	9	3
Papulosquamous disease	1	1
Pigment anomalies	0	1
Pruritus	2	0
Tumors	2	2

### Reliability test of questionnaires

Cronbach’s α coefficients revealed that reliabilities of patients’ responses exceed 0.70, indicating an acceptable level of internal consistency.

### Comparison of the groups

To compare the two independent groups (laboratory tests vs. no laboratory test), an independent *t*-test was conducted. The laboratory test group had a lower positive attitude toward doctors and less intention to follow doctor's recommendations as compared to the no laboratory test groups. However this difference was not significant ( Table 2). In addition, the statistical testing revealed no significant differences in the patient’s, ‘authoritarian personality’, ‘mood’, and ‘intention to accept recommendations’ between the two groups.

**Table 2.** Comparison between groups: results of the unpaired *t*-test (Lab, the laboratory test group; No Lab, no laboratory test group).

Variables	Group	Mean	*p*-value
Attitude toward doctor’s recommendations	Lab No lab	4.060 4.449	0.096
Intention to follow doctor’s recommendations	Lab No lab	4.580 4.890	0.268
Authoritarian personality	Lab No lab	4.115 4.310	0.339
Trust toward doctors	Lab No lab	4.856 4.776	0.268
Positive mood	Lab No lab	3.614 3.426	0.189
Negative mood	Lab No lab	3.046 2.830	0.276

### Moderating effects of patient’s personality

Next, we investigated the effects of variables (laboratory tests vs. no laboratory test, high or low trust toward doctors, and high or low authoritarian personality) on patient attitude toward doctors and intention to accept their recommendations using two-way ANOVA. The groups (laboratory tests vs. no laboratory test) were divided into two groups based on the median value of two variables: trust toward doctors (cut-off value, 4.80) and authoritarian personality (cut-off value, 4.25).

According to the analysis of effects of trust toward doctors, the patients who trust doctors expressed a more positive attitude (3.90 vs. 4.79, *p*<0.001) and an increased intention to follow the doctor’s recommendations (4.28 vs. 5.36, *p*<0.001) compared to patients that had less faith in their doctors. There was no relationship between trust in the doctor’s recommendations and the receipt of laboratory tests ( Table 3).

Analysis of the effects of having an authoritarian personality showed a significant effect of authoritarian personality on both the attitude toward doctor’s recommendations, and the intention to follow the recommendations ( Table 4). Patients with a more authoritarian personality were more positive toward the doctor’s recommendations (3.99 vs. 4.65, *p*=0.01) and had a more intention to follow the recommendation (4.35 vs. 5.24, *p*=0.001). In addition, there was a significant relationship between authoritarian personality and laboratory tests ( Table 4). For patients with a more authoritarian personality, laboratory tests decreased the intention to follow the recommendation (5.74 vs. 4.77, p<0.05).

**Table 3.** Effects of the patient personality ‘trust toward doctors’: results of the two-way analysis of variance (Lab, the laboratory test group; No Lab, no laboratory test group).

Independent variables	Dependent variables		Number of patients	Mean
Attitude toward doctor’s recommendations	Lab	Higher trust† Lower trust‡	24 26	4.389 3.756
	No lab	Higher trust Lower trust	18 32	5.315 4.031
Intention to follow doctor’s recommendations	Lab	Higher trust Lower trust	24 26	5.021 4.173
	No lab	Higher trust Lower trust	18 32	5.806 4.375
			*p*-value	
Attitude toward doctor’s recommendations		Lab Trust Lab x trust	0.017 <0.001 0.189	
Intention to follow doctor’s recommendations		Lab Trust Lab x trust	0.062 <0.001 0.268	

†, ‡ Measure of the ‘Trust toward the doctors’ divided into two groups by median value.

**Table 4.** Effects of the patient personality ‘authoritarian personality’: results of the two-way analysis of variance (Lab, the laboratory test group; No Lab, no laboratory test group; autho, authority personality).

Independent variables	Dependent variables		Number of patients	Mean
Attitude toward doctor’s recommendations	Lab*	Higher autho† Lower autho‡	22 28	4.515 3.702
	No lab**	Higher autho Lower autho	21 29	4.794 4.276
Intention to follow doctor’s recommendations	Lab	Higher autho Lower autho	22 28	4.773 4.429
	No lab	Higher autho Lower autho	21 29	5.738 4.276
			*p*-value	
Attitude toward doctor’s recommendations		Lab Autho Lab x autho	0.097 0.010 0.563	
Intention to follow doctor’s recommendations		Lab Autho Lab x autho	0.125 0.001 0.036	

†, ‡ Measure of the ‘Authoritarian personality’ divided into two groups by median value.

### Effects of gender and age

Additionally, two-way ANOVA and correlation analysis were performed to evaluate variance by sex and age. Males had a more positive attitude toward the doctor’s recommendations (4.72 vs. 3.92, *p*<0.05) and more intention to follow the recommendations (5.06 vs. 4.47, *p*<0.05), as compared to females ( Table 5). Also, measurements of authoritarian personality and trust in doctors were significantly higher in males (authority personality; 4.52 vs. 3.96, *p*<0.01, trust toward doctors; 5.04 vs. 4.64, *p*=0.05). There was no interaction between sex and two personal traits on compliance ( *p*>0.1).

By correlation analysis, intention to follow the doctor’s recommendations and trust toward the recommendation had a tendency to increase in older patients (correlation coefficient was 0.374 ( *p*<0.001) and 0.432 ( *p*<0.001), respectively ( Table 6).

**Table 5.** Effects of gender: results of the two-way analysis of variance.

Independent variables	Dependent variables		Number of patients	Mean
Attitude toward doctor’s recommendations	Lab*	Male Female	22 28	4.409 3.786
	No lab**	Male Female	23 27	5.015 4.049
Intention to follow doctor’s recommendations	Lab	Male Female	22 28	4.750 4.446
	No lab	Male Female	23 27	5.348 4.500
			*p*-value	
Attitude toward doctor’s recommendations		Lab Gender Gender x lab	0.084 0.002 0.494	
Intention to follow doctor’s recommendations		Lab Gender Gender x lab	0.239 0.039 0.325	

* Laboratory test group.

** No laboratory test group.

**Table 6.** Effects of age: results of the correlation analysis.

	Mean	Pearson correlation coefficient	*p*-value
Age	38.490		
Attitude toward doctor’s recommendations	4.277	0.374	<0.001
Intention to follow doctor’s recommendations	4.735	0.432	<0.001

### Discussion

Compliance of a patient is ‘the extent to which the patient’s behavior matches agreed recommendation from the prescribers’ ^19^. It is critical element in determining the beneficial effect of medical care and decided by interaction of multiple factors ^20, 21^. Many psychological models including Becker’s health belief model have been introduced to explain compliance ^9^. According to them, patients show increased compliance when they believe the threat of disease will be diminished by following health recommendations ^22^. Also the doctor’s attitude towards the patient, doctor’s ability to bring out and respect the patient’s concerns, ability to provide appropriate information, and ability to show empathy are important factors of compliance ^23^.

Although the psychological aspects of patients and doctors have been emphasized in patient compliance, most of compliance studies in dermatology have focused on the diagnosis and treatment options as determining factors ^24– 26^. In 2010, Kim *et al.* ^8^ first described the psychological impacts of skin biopsy, which is one of the most frequently performed diagnostic tools in dermatology. This study showed that skin biopsy subconsciously increase patients’ positive attitude toward doctor’s recommendations and potentially bias patients’ decision for the treatment. This phenomenon was explained by terror management theory (TMT), which is generally studied in psychology and marketing, but also has been applied to medicine ^27^. TMT states that people behave to protect themselves from fear of death when they are reminded of their own deaths. Societies traditionally have built cultural worldview (such as laws, cultures, and religions), and individuals adhere to these cultural worldview when confronting fear of mortality ^10^. In medicine, certain health behaviors such as breast self-exam can subconsciously activate and facilitate people to remind death, and death act as conditioning behavior towards a longer life ^27^.

In this study, we hypothesized that the laboratory tests, which are frequently recommended in dermatologic outpatient clinic, could be a stimulant of mortality salience because it reminds patients of more severe diseases or vital organ dysfunctions. Based on TMT, we expected that patients who had laboratory tests adhere to doctor’s recommendations more than the patients who did not have laboratory tests.

Interestingly, the result of this study was contrary to our expectation. Although the differences between two groups were not statistically significant, the patients who had laboratory tests had tendency to show even lower positive attitude toward the doctor’s recommendations and less intention to follow the recommendations. This suggests that laboratory tests do not subliminally increase the patient’s fear of their disease or increase compliance.

Despite statistically insignificance in this study, no relationship between laboratory tests and adherence to doctors’ recommendation raises important implications for future research. For example, biomarkers for guiding therapy selection and disease monitoring have been developed with skin biopsy in the field of dermatology ^28^. However, skin biopsy has a risk of subliminally increase the patient’s fear or anxiety and subconsciously bias patients’ decision for the treatment ^8^. On the other hand, laboratory tests such as blood test and urine test do not influence patient compliance and supply a neutral circumstance for patients’ treatment decisions. Therefore, this study implicates that laboratory tests could be superior to skin biopsy as a source of biomarker development.

The limitation of this study is that we assessed patient compliance as a hypothetical construct and psychological concepts were examined using an indirect method. Also, the small sample size limited the statistical power of this study, and also the diagnosis of the participants and the types of treatments could not be randomized between groups.

We failed to prove our hypothesis based on TMT, but the results still have important implications in dermatologic outpatient clinics. The performance of laboratory tests does not seem to reinforce the patient’s fear of their own disease, and instead possibly decreases lower the patient’s trust in doctors. Doctors should not hesitate to perform laboratory tests when the situation requires this, but we suggest that careful consideration about possible psychological impacts is needed when ordering laboratory tests for patients. To enhance patient compliance with doctor’s recommendations, doctors should carefully consider not only their diagnosis or treatment plan, but also the psychological impact of commonly performed tests and the patient’s personality.

Data for Laboratory Tests and Compliance of Dermatologic Outpatients The scores of each question in the group of 50 patients who did not have any laboratory tests
The individual scores of each question are marked in a seven-point scale. The intention of each question and each patient's demographic information are also indicated.
The scores of each question in the group of 50 patients who underwent laboratory tests
The individual scores of each question are marked in a seven-point scale. The intention of each question and each patient's demographic information are also indicated.
The questionnaire for the study
The questionnaire is developed to measure trust towards doctors, authoritarian personality, current mood, attitude toward the doctor’s recommendation, and intention to follow the recommendations. The original questionnaire in Korean is translated into English.
