# Supplementary material for: Laboratory tests and compliance of dermatologic outpatients
Source: F1000Res. 2013 Oct 7;2:206. [Version 1] doi: 10.12688/f1000research.2-206.v1 (PMC3869483; doi:10.12688/f1000research.2-206.v1)
Supplement: Data for Laboratory Tests and Compliance of Dermatologic Outpatients — The scores of each question in the group of 50 patients who did not have any laboratory tests The individual scores of each question are marked in a seven-point scale. The intention of each question and each patient's demographic information are also indicated. The scores of each question in the group of 50 patients who underwent laboratory tests The individual scores of each question are marked in a seven-point scale. The intention of each question and each patient's demographic information are also indicated. The questionnaire for the study The questionnaire is developed to measure trust towards doctors, authoritarian personality, current mood, attitude toward the doctor’s recommendation, and intention to follow the recommendations. The original questionnaire in Korean is translated into English. [file f1000research-2-2244-s0000.tgz › 3._Survey_translated_into_English.docx]

**Survey**

(translated from Korean into English)

This survey is designed to investigate your opinion on the medical services of this hospital and the hospital itself in order to create an improved medical care environment. There is no right or wrong answer to each question. We just ask you to answer the questions honestly what you have thought usually.

The records to obtain your identities are not collected. The collected data will be handled anonymously for statistics, and will not be opened individually.

We greatly appreciate your precious time and important input to this survey.

**Read the questions below and check how you agree to each question**

|  | **Strongly disagree** | **Disagree** | **Disagree Somewhat** | **Undecided** | **Agree Somewhat** | **Agree** | **Strongly agree** |
| --- | --- | --- | --- | --- | --- | --- | --- |
| **1. I believe that doctors put hospitals profit before the health of patients** |  |  |  |  |  |  |  |
| **2. I believe that doctors keep patients’ personal information confidential** |  |  |  |  |  |  |  |
| **3. I trust the doctors’ judgment when it comes to my medical care** |  |  |  |  |  |  |  |
| **4. I believe that doctors perform necessary medical tests and procedures regardless of the cost** |  |  |  |  |  |  |  |
| **5. I believe that doctors perform only medically required tests and procedures** |  |  |  |  |  |  |  |

**Read the questions below and check how you agree to each question**

|  | **Strongly disagree** | **Disagree** | **Disagree Somewhat** | **Undecided** | **Agree Somewhat** | **Agree** | **Strongly agree** |
| --- | --- | --- | --- | --- | --- | --- | --- |
| **6. It’s fun bearing danger** |  |  |  |  |  |  |  |
| **7. I sometimes do dangerous things for fun** |  |  |  |  |  |  |  |
| **8. I like friends with unpredictable behaviors** |  |  |  |  |  |  |  |

**Read the questions below and check how you agree to each question**

|  | **Strongly disagree** | **Disagree** | **Disagree Somewhat** | **Undecided** | **Agree Somewhat** | **Agree** | **Strongly agree** |
| --- | --- | --- | --- | --- | --- | --- | --- |
| **9. In various gatherings I can talk or do actions to amuse people** |  |  |  |  |  |  |  |
| **10. I can sometimes make up words to make people happy** |  |  |  |  |  |  |  |
| **11. I can act or behave as if I am an actor/actress** |  |  |  |  |  |  |  |
| **12. I can hide my true self** |  |  |  |  |  |  |  |
| **13. I sometimes change my ordinary behavior patterns in order to suit other people’s feelings or situations** |  |  |  |  |  |  |  |

**Read the questions below and check how you agree to each question**

|  | **Strongly disagree** | **Disagree** | **Disagree Somewhat** | **Undecided** | **Agree Somewhat** | **Agree** | **Strongly agree** |
| --- | --- | --- | --- | --- | --- | --- | --- |
| **14. A strict control and order is required for efficiency of work** |  |  |  |  |  |  |  |
| **15. The most important virtue that a child has to learn is to obey and respect elders** |  |  |  |  |  |  |  |
| **16. A great leader makes the era** |  |  |  |  |  |  |  |
| **17. A person who follows the words of the superior is a good person** |  |  |  |  |  |  |  |
| **18. I do not change my ordinary behavior patterns in order to suit other people’s feelings or situations** |  |  |  |  |  |  |  |

**Read the questions below and check how you agree to each question**

|  | **Strongly disagree** | **Disagree** | **Disagree Somewhat** | **Undecided** | **Agree Somewhat** | **Agree** | **Strongly agree** |
| --- | --- | --- | --- | --- | --- | --- | --- |
| **19. The emotion that I feel now is “Interested”** |  |  |  |  |  |  |  |
| **20. The emotion that I feel now is “Attentive”** |  |  |  |  |  |  |  |
| **21. The emotion that I feel now is “Excited”** |  |  |  |  |  |  |  |
| **22. The emotion that I feel now is “Inspired”** |  |  |  |  |  |  |  |
| **23. The emotion that I feel now is “Strong”** |  |  |  |  |  |  |  |
| **24. The emotion that I feel now is “Alert”** |  |  |  |  |  |  |  |
| **25. The emotion that I feel now is “Enthusiastic”** |  |  |  |  |  |  |  |
| **26. The emotion that I feel now is “Active”** |  |  |  |  |  |  |  |
| **27. The emotion that I feel now is “Pride”** |  |  |  |  |  |  |  |
| **28. The emotion that I feel now is “Determined”** |  |  |  |  |  |  |  |
| **29. The emotion that I feel now is “Irritable’** |  |  |  |  |  |  |  |
| **30. The emotion that I feel now is “Distressed”** |  |  |  |  |  |  |  |
| **31. The emotion that I feel now is “Ashamed”** |  |  |  |  |  |  |  |
| **32. The emotion that I feel now is “Upset”** |  |  |  |  |  |  |  |
| **33. The emotion that I feel now is “Jittery”** |  |  |  |  |  |  |  |
| **34. The emotion that I feel now is “Guilty”** |  |  |  |  |  |  |  |
| **35. The emotion that I feel now is “Scared”** |  |  |  |  |  |  |  |
| **36. The emotion that I feel now is “Hostile”** |  |  |  |  |  |  |  |
| **37. The emotion that I feel now is “Nervous”** |  |  |  |  |  |  |  |
| **38. The emotion that I feel now is “Afraid”** |  |  |  |  |  |  |  |

**Read the following story and answer to the questions below. Even if the situation presented is different from reality, suppose you are in the same situation and answer the questions.**

**You have a severe and chronic skin disease, and your doctor recommends a newly developed treatment method for you. He tells you that this method can reduce treatment time, but it is more expensive than traditional treatment and safety is not fully guaranteed.**

**Read the questions below and check how you agree to each question**

|  | **Strongly disagree** | **Disagree** | **Disagree Somewhat** | **Undecided** | **Agree Somewhat** | **Agree** | **Strongly agree** |
| --- | --- | --- | --- | --- | --- | --- | --- |
| **39. I am favorable to the doctor’s suggestions** |  |  |  |  |  |  |  |
| **40. I think well of the doctor’s suggestions** |  |  |  |  |  |  |  |
| **41. The doctor’s suggestions are appealing** |  |  |  |  |  |  |  |

**Read the questions below and check how you agree to each question**

|  | **Strongly disagree** | **Disagree** | **Disagree Somewhat** | **Undecided** | **Agree Somewhat** | **Agree** | **Strongly agree** |
| --- | --- | --- | --- | --- | --- | --- | --- |
| **42. I will follow the recommendations of the doctor** |  |  |  |  |  |  |  |
| **43. There is a possibility of following recommendations of the doctor** |  |  |  |  |  |  |  |
