# Supplementary material for: Laboratory tests and compliance of dermatologic outpatients
Source: F1000Res. 2013 Oct 7;2:206. [Version 1] doi: 10.12688/f1000research.2-206.v1 (PMC3869483; doi:10.12688/f1000research.2-206.v1)
Supplement: Peer review history

# Peer review: Referee response for version 1

**Published:** 2013-11-01

**Authors:** Roberto Arenas

**Affiliations:** 1 Departamento of Dermatology, Hospital General Dr Manuel Gea Gonzalez, Tlalpan, DF 14000, Mexico

This article is interesting, practical and useful

I have read this submission. I believe that I have an appropriate level of expertise to confirm that it is of an acceptable scientific standard.

# Peer review: Referee response for version 1

**Published:** 2013-10-28

**Authors:** Mohamed Badawy Abdel-Naser

**Affiliations:** 1 Department of Dermatology and Venereology, Ain Shams University, Cairo, Egypt

The manuscript is well written and informative. However, there some remarks in the following sections that could be be taken into consideration:

**Introduction:**

- *“In a dermatologic clinic, laboratory tests such as blood and urine tests are one of the main examinations* *”* Is it better to use "investigations" or "analyses" instead of repeating the word "tests" likewise the second “the cultural worldviews” can be replaced by “them”
- *“..blood urea nitrogen analysis, and creatinine analysis.*” is it fine with “blood urea nitrogen and creatinine analyses” 

**Survey:**

- “towards” and “toward” are both used in the whole manuscript. You may adhere to one or same spelling

**Trust toward doctors:**

- “.. *believe that doctors perform: 1. Perform only medically necessary*…” you may delete the first “perform”.
- “ *In case of fourth state reverse scale was used, and average of patients’ scale to these five questions was used*” please revise this statement.

**Authoritarian personality:**

- *“..amount of trust towards doctors,might influences”* consider “might influence”
- “ *..the scale consist*” consider “the scale consists”

**Reliability test of questionnaires:**

- “ *Cronbach’s α coefficients*” this statistical method has not been mentioned or referred to in the statistics section

**Comparison of the groups:**

- “.. *an independent t-test*” is it equivalent to unpaired t test that has been mentioned in the statistics section? You should adhere to the same terminology. 

**Moderating effects of patient’s personality:**

- “ *The patients who trust doctors expressed a more positive attitude (3.90 vs. 4.79, p<0.001)*” Please check these values with those in the table for errors. This is also applicable to all values mentioned in the text with their corresponding values in the tables. Also the arrangement of the values (higher values and then the lower).

**Discussion:**

- “... *skin biopsy subconsciously increase patients*” consider “increases” also in other locations in the section.
- “ .. *and death act as conditioning*” consider “acts”
- " *Despite statistically insignificance in this study, no relationship between laboratory tests and adherence to doctors’ recommendation raises important implications for future research*” Please revise this statement
- “.. *instead possibly decreases lower the patient’s trust in doctors*” please revise the statement

I have read this submission. I believe that I have an appropriate level of expertise to confirm that it is of an acceptable scientific standard.
